# A simple and effective screening strategy for early multiple system atrophy diagnosis and α-Synuclein forms in erythrocytes

**DOI:** 10.3389/fnagi.2025.1533504

**Published:** 2025-02-21

**Authors:** Ying Jiang, Jianing Jin, Yingshan Piao, Yixuan Yin, Lian Tang, Qixuan Guan, Yang Gao, Tao Feng, Zhan Wang

**Affiliations:** ^1^Department of Neurology, Center for Movement Disorders, Beijing Tiantan Hospital, Capital Medical University, Beijing, China; ^2^China National Clinical Research Center for Neurological Diseases, Beijing, China; ^3^Department of Neurology, Affiliated Hospital of Qingdao University, Qingdao, China; ^4^Department of Neurology, Beijing Tiantan Hospital, Capital Medical University, Beijing, China; ^5^Department of Neurology, Qian 'an Yanshan Hospital, Hebei, China

**Keywords:** differential diagnosis, possible multiple system atrophy, early-stage PD, α-Synuclein, post-void residual urine volume

## Abstract

**Background:**

Urinary dysfunction is an early manifestation of autonomic dysfunction in Multiple System Atrophy (MSA) and often precedes orthostatic hypotension. This study investigated the diagnostic efficacy of post-void residual (PVR) urine volume in differentiating possible MSA from early-stage Parkinson’s disease (PD) and sought to identify a feasible combination of autonomic nervous system indicators for clinical use. The distribution of α-Synuclein (α-Syn) forms in erythrocyte was preliminary explored.

**Methods:**

This study included 70 patients with MSA-P, 73 with MSA-C, and 71 with PD. All participants underwent assessments including bladder residual urine ultrasound, the supine-to-standing test (STS), external anal sphincter electromyography (EAS-EMG), brain MRI, Mini-Mental State Examination (MMSE), and Montreal Cognitive Assessment (MoCA). Receiver operating characteristic (ROC) curves determined the diagnostic value of PVR urine volume and other autonomic indicators for possible MSA. Immunofluorescence staining of α-Syn forms in red blood cells (RBCs) was also performed.

**Results:**

PVR urine volume, ΔSBP and ΔDBP (at 1 and 3 min), and EAS-EMG parameters were significantly increased in MSA-P and MSA-C patients compared to PD (*p* < 0.01), with similar differences observed between possible MSA-P/MSA-C and early-stage PD patients. ROC analysis showed that PVR urine volume had diagnostic value in differentiating possible MSA-P (AUC = 0.668, cut-off 24.5 mL) and MSA-C (AUC = 0.759, cut-off 47.5 mL) form early-stage PD patients. ΔSBP at 1 and 3 min also distinguished possible MSA-P (AUC = 0.702, 0.730) and MSA-C (AUC = 0.707, 0.718) from early-stage PD. Combining PVR urine volume and ΔSBP (at 1 and 3 min) further improved diagnostic accuracy, with an AUC of 0.817 (sensitivity 57.1%, specificity 96.8%) for possible MSA-P and an AUC of 0.794 (sensitivity 68.6%, specificity 87.1%) for MSA-C from early-stage PD. On a molecular level, oligo-α-Syn predominantly localized to RBC membrane fractions in MSA patients, while α-Syn pS129 was primarily detected in the RBC cytoplasm of PD patients.

**Conclusion:**

Combining PVR urine volume and ΔSBP (at 1 and 3 min) is an easily accessible and effective method for distinguishing possible MSA from early-stage PD. This combination should be considered for routine assessment in Parkinsonism. Distinct α-Syn forms distribution in erythrocytes could be considered as a useful biomarker for differential diagnosis.

## 1 Introduction

Multiple system atrophy (MSA) is an adult-onset, sporadic, and rapidly progressive neurodegenerative disorder characterized by the pathological aggregation of insoluble α-synuclein (α-syn) predominantly in oligodendrocytes (Miki et al., 2019). The clinical manifestation of MSA primarily encompasses autonomic dysfunction, poorly levodopa-responsive parkinsonism, and cerebellar ataxia (McKay and Cheshire, 2018). The early stages of MSA often resemble Parkinson’s disease (PD), posing diagnostic challenges that can lead to delays in accurate identification and management. Notably, autonomic dysfunction may be one of the earliest manifestations of MSA. Previous research indicates that 73% of individuals with MSA present with autonomic dysfunction as their initial symptom, compared to only 3% with initial motor symptoms (Yamamoto et al., 2014). A longitudinal study by Kaufmann et al. followed 100 patients with isolated autonomic dysfunction, revealing that 8% ultimately progressed to MSA (Gilman et al., 2008). Importantly, autonomic disturbances are present in nearly half of all patients early in the disease course and progressively worsen as MSA advances. Consequently, there is growing interest in utilizing comprehensive autonomic assessments to differentiate MSA from PD.

Generally, dysautonomia in MSA included marked orthostatic hypotension and urinary incontinence. The second consensus diagnostic criteria for MSA, published in 2008, recognizes orthostatic hypotension, impotence in males, and urinary incontinence or retention as parts of the consensus criteria (Ogawa et al., 2017). Urinary dysfunction frequently emerges early in the course of MSA, rapidly progressing to significant post-void residual (PVR) volume and urinary retention, severely impacting a patient’s quality of life. Prevalence studies indicate that at least 90% of MSA patients experience lower urinary tract symptoms, which are more prevalent and severe than in PD patients (Sakakibara et al., 2018). It is estimated that 33–100% of MSA patients exhibit bladder overactivity, and 33% present with uninhibited external sphincter relaxation during the filling phase (Sakakibara et al., 2001). Incomplete bladder emptying is also common, with approximately 47% of MSA patients demonstrating a PVR urine volume exceeding 100 mL (Jellinger, 2020). Sakakibara et al. demonstrated that urinary symptoms are often the earliest and most common autonomic feature in MSA, preceding even orthostatic hypotension (Sakakibara et al., 2018). Difficulty voiding, the most frequent urinary symptom, occurs in nearly 79% of MSA patients. Furthermore, approximately 60% of individuals with MSA experience urinary symptoms prior to or concurrent with motor disturbances, increasing the risk of misdiagnosis (Sakakibara et al., 2000). Despite the high prevalence of urinary dysfunction in MSA, research on this aspect remains limited, underscoring the critical need to delineate the clinical characteristics of urinary dysfunction in MSA and PD.

MSA is classified into two phenotypes Parkinsonian (MSA-P) and cerebellar entity (MSA-C) based on the predominant motor features at onset. According to the latest MSA criteria formulated by Movement Disorders Society Scientific Issues Committee (Wenning et al., 2022), the PVR urine volume was considered as an important part consensus criteria of dysautonomia, but the specific values in “clinically probable” or “possible” MSA (criteria for MSA published in 2008) is not recommended. Additionally, research investigating differences in PVR urine volume between possible MSA and early-stage PD remains sparse. To our knowledge, no studies have comprehensively evaluated the utility of PVR urine volume, in conjunction with other autonomic dysfunction indicators, to differentiate possible MSA from early-stage PD.

External anal sphincter electromyography (EAS-EMG) has been recognized as a valuable tool for assessing autonomic function and aiding in the diagnosis of MSA (Cao et al., 2020). However, its ability to reliably differentiate MSA from PD and the correlation between bladder dysfunction and EAS-EMG alterations in MSA remain subjects of ongoing debate Furthermore, the moderately invasive nature of EAS-EMG may be poorly tolerated by some patients. Therefore, there is a pressing need to explore novel, less invasive diagnostic strategies for distinguishing possible MSA from early-stage PD.

Accumulating evidences have shown that different α-Syn forms in body fluids, such as blood (Li et al., 2021), cerebrospinal fluid (CSF) (Barkovits et al., 2020), and plasma (Jonsson et al., 2013), hold promise as diagnostic biomarkers for PD and MSA. Shi et al. observed decreased α-syn levels in the CSF of both PD and MSA patients, with a more pronounced decrease in MSA (Shi et al., 2011). Another study reported lower plasma α-syn concentrations in MSA patients compared to PD patients (Lee et al., 2006). In erythrocytes, several studies have detected that the levels of α-Syn forms in PD and MSA patients were increased compared with those in healthy controls (Liu et al., 2019; Tian et al., 2019). However, due to the hemolysis contamination, the α-syn results based on blood were confounded. Therefore, direct detection of α-Syn, oilg-α-Syn, and α-Syn pS129 concentrations in erythrocyte might be an appropriate method in order to avoid hemolysis interference.

In the present study, we aimed to (1) investigate differences in PVR urine volume and other autonomic dysfunction indicators between possible MSA and early-stage PD; (2) explore the diagnostic accuracy of readily accessible screening indicators, alone and in combination, for early-stage disease differentiation; and (3) preliminarily detect the α-Syn forms distribution in erythrocytes between the two diseases and health controls.

## 2 Methods

### 2.1 Subjects and ethics statement

This study included 143 patients with probable or possible MSA and 71 patients with PD erythrocyte University, between December 2019 and August 2024. All participants provided informed consent. We adopted the Movement Disorder Society (MDS) Clinical Diagnostic Criteria for PD (Postuma et al., 2015). MSA diagnoses adhered to the second consensus criteria, published in 2008. According to previous researches, H-Y staging ≤2.5 is considered to be the early stage of PD (Gao et al., 2018; Chen et al., 2016). Two experienced neurologists specializing in neurodegenerative diseases confirmed the diagnoses of PD and MSA. The Ethics Committee at Beijing Tiantan Hospital approved this research, which was conducted in accordance with the Declaration of Helsinki.

### 2.2 Evaluation and datum collection

Demographic variables (age, sex) and clinical characteristics were collected for all participants. Each underwent an evaluation including disease duration, renal function, medical history, neurological examinations, levodopa equivalent daily dose (LEDD), and sonographic PVR urine volume measurements. All patients underwent brain magnetic resonance imaging (MRI) to aid in the differential diagnosis. PD patients were also assessed using the Unified Parkinson’s Disease Rating Scale (UPDRS)-III and Hoehn and Yahr (H&Y) stage. Non-motor symptoms, including blood pressure and heart rate changes during the supine-to-standing test (STS), cognition, constipation, and rapid eye movement sleep behavior disorder (RBD), were also evaluated. Cognitive status was assessed using the Mini-Mental State Examination (MMSE) and Montreal Cognitive Assessment (MoCA).

PVR urine volume was measured using a LOGIQ E9 ultrasound device with XD clear (GE, Boston, Massachusetts, United States). Bladder volume was calculated based on the bladder’s outline using ultrasound echo signals, with a measurement range of 0 to 999 mL and an accuracy of ±20% or ±20 mL.

All patients underwent EAS-EMG using a Nicolet EDX electromyographic evoked potentiometer, performed by experienced neurologists and EMG technicians. Data analysis included duration, amplitude, phase, polyphase wave ratio, and satellite potential occurrence rate of EAS-EMG motor unit potentials (MUPs).

### 2.3 Erythrocyte collection and immunofluorescence

Venous blood samples (5 mL) were collected from 10 MSA-P, 10 MSA-C, and 10 PD patients into K2-EDTA Vacutainers (367863, BD, Franklin Lakes, NJ, United States) and processed within 2 h. Red blood cells (RBCs) were isolated from whole blood by centrifugation at 1,500 × g for 10 min at 4°C.

The RBCs (2 mL) were transferred to a new tube, washed with phosphate-buffered saline (PBS), and centrifuged again at 3,000 × g for 10 min at 4°C. The supernatant was discarded, and the washed RBCs were diluted with PBS. RBC smears were then prepared using a cytospin (CYTOSPIN IV, AHSI, Italy) by centrifugation at 700 rpm for 6 min.

The RBCs were fixed with methanol at room temperature for 20 min and then permeabilized with 0.1% Triton X-100. After blocking with 5% bovine serum albumin (Sigma, Poole, UK) for 1 h, the RBCs were incubated overnight at 4°C with the following primary antibodies: MJFR1 (ab138501, Abcam, Cambridge, MA, United States; diluted 1:200) for α-Syn detection, MJFR-14-6-4-2 (ab209538, Abcam; diluted 1:200) for α-Syn aggregates (This antibody is reported to recognize oligomeric forms of α-Syn), and a pS129 antibody (cat825701, BioLegend, San Diego, CA, United States; diluted 1:200). The slides were then rinsed and incubated for 1.5 h at room temperature with Alexa-conjugated secondary antibodies: donkey anti-rabbit Alexa Fluor 488 (ab150077, Abcam; diluted 1:200), donkey anti-rabbit Alexa Fluor 647 (ab150075, Abcam; diluted 1:200) and donkey anti-mouse Alexa Fluor 647 (ab150107, Abcam; diluted 1:200). Finally, the slides were incubated with the Zenon^™^ Alexa Fluor 405 mouse IgG1 labeling kit (Z25013, Life Technologies, Eugene, OR, United States) to label antibodies against CD235a on the erythrocyte membrane. Slides were observed under a Zeiss LSM 700 confocal microscope (Carl Zeiss Microscopy GmbH, Germany) using a 40 × objective.

### 2.4 Data analysis and statistics

Statistical analyses were performed using SPSS 26.0 software (SPSS Inc., Chicago, IL, United States). Graphs were generated using Prism 7.0 (GraphPad software, La Jolla, CA, United States). Continuous variables with normal distribution were presented as mean ± standard deviation (SD), and those with non-normal distribution as median (interquartile range). The *t*-test and the chi-square test were used to compare differences in clinical data between groups. The Mann–Whitney U test was used for non-normally distributed data between two groups. The Kruskal–Wallis test was assessed for non-normally distributed data among three groups. The diagnostic value of PVR urine volume, ΔSBP (at 1 and 3 min) and EMG parameters were investigated using receiver operating characteristic (ROC) curves, and the areas under the curve (AUC) were calculated. A two-sided *p*-value < 0.05 was considered statistically significant.

## 3 Results

### 3.1 Demographic and clinical characteristics

A total of 214 patients (70 with MSA-P, 73 with MSA-C, and 71 with PD) met the probable or possible criteria for MSA or PD. The Kruskal-Wallis test revealed significant group differences in PVR urine volume, disease duration, 1-min and 3-min ΔSBP and ΔDBP, constipation percentage, RBD percentage, average duration, amplitude, polyphasity, phases, satellite potential occurrence rate of EAS-EMG, and daily levodopa equivalent dose (LEDD). *Post hoc* comparisons (Mann–Whitney U test) showed that most of these differences were also significant between MSA-C versus PD (*p* < 0.05) and MSA-P versus PD (*p* < 0.05). No significant differences were observed in gender, age, prostate indicators, MMSE, MoCA, 1-min ΔHR, 3-min ΔHR, estimated glomerular filtration rate (eGFR), or the percentages of patients with diabetes, nephropathy, hypertension, smoking, or alcohol consumption among the MSA-P, MSA-C, and PD groups. Demographic and clinical characteristics are summarized in Table 1.

**Table 1:** Demographic and clinical characteristics of all participants.

Characteristics	MSA-P (*n* = 70)	MSA-C (*n* = 73)	PD (*n* = 71)	*p*	P MSA-PvsPD	MSA-CvsPD
Females/males	26/44	38/35	40/31	0.057	0.022	0.606
Age, years	61.50 (56.00–68.00)	61.00 (54.00–67.00)	64.00 (55.00–67.00)	0.506	0.579	0.286
Duration, years	3.00 (2.00–4.00)	2.00 (1.00–3.50)	6.00 (4.00–9.00)	<0.001	<0.001	<0.001
Prostate	3.40 (2.85–4.00) 4.30 (4.05–4.80) 3.47 ± 0.81	3.50 (3.05–4.35) 4.20 (3.25–4.55) 3.12 ± 0.61	3.20 (3.00–3.80) 4.30 (4.00–5.00) 3.33 ± 0.66	0.624 0.224 0.183	0.845 0.797 0.49	0.358 0.104 0.236
PVR curine volume, ml	140.33 (8.25–159.25)	99.52 (11.5–149.5)	5.00 (3.50–20.00)	<0.001	<0.001	<0.001
MMSE	27.00 (23.00–28.00)	27.00 (24.00–28.00)	28.00 (25.00–29.00)	0.170	0.042	0.143
Moca	21.00 (17.00–25.00)	21.00 (17.50–24.00)	21.00 (18.00–26.00)	0.341	0.493	0.123
1 min ΔSBP, mmHg	16.00 (5.50–28.00)	20.00 (9.00–31.00)	3.00 (−7.00 to 12.00)	<0.001	<0.001	<0.001
1 min ΔDBP, mmHg	3.00 (−1.50 to 10.00)	8.00 (−1.00 to 14.50)	−1.00 (−8.00 to 4.00)	<0.001	<0.001	<0.001
1 min ΔHR	−7.00 (−11.00 to −3.00)	−8.00 (−12.5 to −4.00)	−6.00 (−10.00 to −1.00)	0.083	0.116	0.031
3 min ΔSBP, mmHg	15.00 (4.00–26.00)	18.00 (8.00–30.00)	2.00 (−5.00 to 9.00)	<0.001	<0.001	<0.001
3 min ΔDBP, mmHg	5.00 (−1.50 to 10.00)	7.00 (−2.00 to 15.00)	−2.00 (−7.00 to 3.00)	<0.001	<0.001	<0.001
3 min ΔHR	−6.00 (−10.00 to −2.5)	−6.00 (−12.50 to −3.50)	−6.00 (−10.00 to −1.00)	0.431	0.567	0.174
eGFR, ml/min	108.40 (102.77–114.45)	112.27 (103.62–118.27)	107.65 (101.60–115.20)	0.155	0.829	0.145
Constipation %	81.43	69.86	57.75	0.009	0.002	0.13
Diabetes %	10.00	13.70	18.31	0.363	0.157	0.450
Nephropathy %	5.71	27.40	16.90	0.1	0.036	0.764
Hypertension %	28.57	30.14	38.03	0.434	0.234	0.318
RBD %	77.14	76.71	40.85	<0.001	<0.001	<0.001
Smoking %	20.00	27.40	28.17	0.467	0.257	0.918
Drinking %	24.29	30.14	21.13	0.450	0.654	0.216
LEDD, mg	649.00 (468.75–956.75)	200.00 (100.00–350.00)	786.00 (600.00–1073.00)	<0.001	0.011	<0.001
Duration (ms)	13.10 (11.90–13.90)	12.50 (11.00–14.00)	10.80 (10.10–12.00)	<0.001	<0.001	<0.001
Amplitude (μv)	574.50 (465.50–680.50)	610.00 (518.00–728.00)	547.00 (436.00–638.00)	0.007	0.002	0.276
Phases	4.80 (4.08–5.53)	4.80 (4.20–5.80)	4.10 (3.60–4.60)	<0.001	<0.001	<0.001
Polyphasity (%)	45.00 (30.00–65.00)	50.00 (35.00–65.00)	40.00 (20.00–50.00)	<0.001	<0.001	0.007
Percentage of satellite potentials (%)	10.00 (5.00–16.78)	7.70 (5.00–15.00)	5.00 (0.00–10.00)	<0.001	0.001	<0.001

Data are shown as the mean (SD) for normally distributed continuous variables, median (IQR) for nonnormally distributed continuous variables, nd *n* (%) for categorical variables. ΔSBP: changes in systolic blood pressure during the supine-to-standing test. ΔDBP: changes in diastolic blood pressure during the supine-to-standing test.

MMSE, Mini-Mental State Examination; MoCA, Montreal Cognitive Assessment; PVR, post-void residual; LEDD, levodopa equivalent daily dose; MSA, Multiple system atrophy; PD, Parkinson’s Disease.

### 3.2 Evaluation of autonomic dysfunction in possible MSA-P, possible MSA-C, and early-stage PD

**Figure 1:**
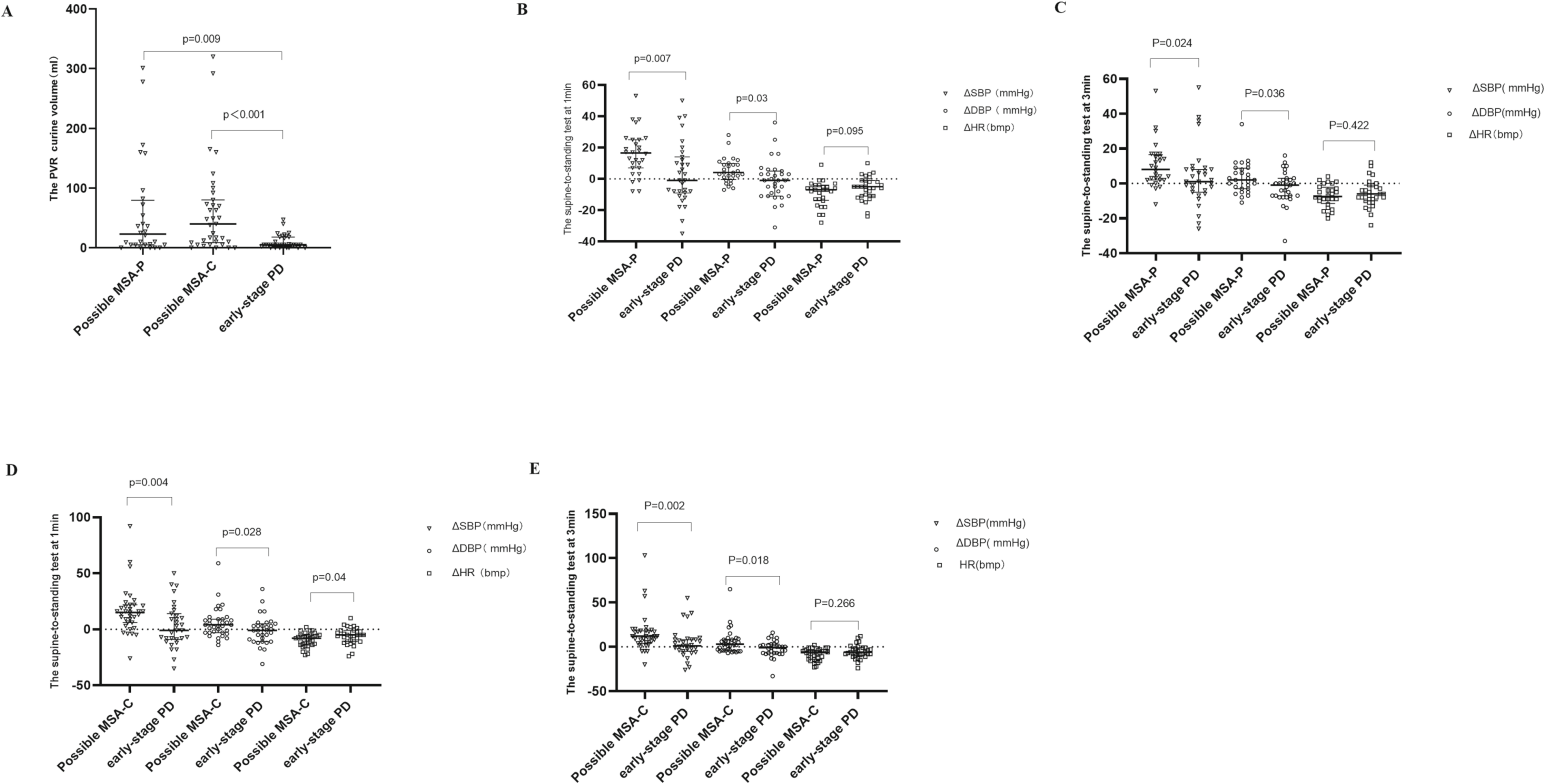
The PVR urine volume and changes of blood pressure and heart rate during STS test in possible MSA-P, possible MSA-C and early-stage PD participants. STS test: supine-to-standing test. ΔSBP: changes in systolic blood pressure during the supine-to-standing test. ΔDBP: changes in diastolic blood pressure during the supine-to-standing test. This scatter plot depicts the median and interquartile range. Long horizontal lines represent median values. **(A)** The PVR urine volume in Possible MSA-P, Possible MSA-C, and Early-Stage PD Participants. **(B)** The changes of blood pressure and heart rate during STS test between possible MSA-P and early-stage PD at 1 min. **(C)** The changes of blood pressure and heart rate during STS test between possible MSA-P and early-stage PD at 3 min. **(D)** The changes of blood pressure and heart rate during STS test between possible MSA-C and early-stage PD at 1 min. **(E)** The changes of blood pressure and heart rate during STS test between possible MSA-C and early-stage PD at 3 min.

According to the second consensus statement on MSA diagnosis, 80 patients met the criteria for probable MSA, and 63 for possible MSA. These were subdivided into MSA-C (38 probable, 35 possible) and MSA-P (42 probable, 28 possible). Given that urinary dysfunction is an early feature in MSA, we investigated PVR urine volume differences between early-stage PD and possible MSA. As expected, PVR urine volume was significantly higher in possible MSA-C and MSA-P patients compared to early-stage PD patients (*p* < 0.001, *p* = 0.009), suggesting its potential as an effective indicator for early differential diagnosis (Figure 1A).

Similarly, we evaluated ΔSBP, ΔDBP, and ΔHR during the STS at 1 and 3 min. Compared to early-stage PD patients, both ΔSBP and ΔDBP were significantly higher in possible MSA-P (Figure 1B) and MSA-C (Figure 1D) patients at 1 min (possible MSA-C ΔSBP *p* = 0.004, ΔDBP *p* = 0.028; possible MSA-P ΔSBP *p* = 0.007, ΔDBP *p* = 0.03) and 3 min [possible MSA-P ΔSBP *p* = 0.024, ΔDBP *p* = 0.036 (Figure 1C); possible MSA-C ΔSBP *p* = 0.002, ΔDBP *p* = 0.018 (Figure 1E)]. No significant differences in ΔHR were observed at 3 min (*p* > 0.05).

**Figure 2:**
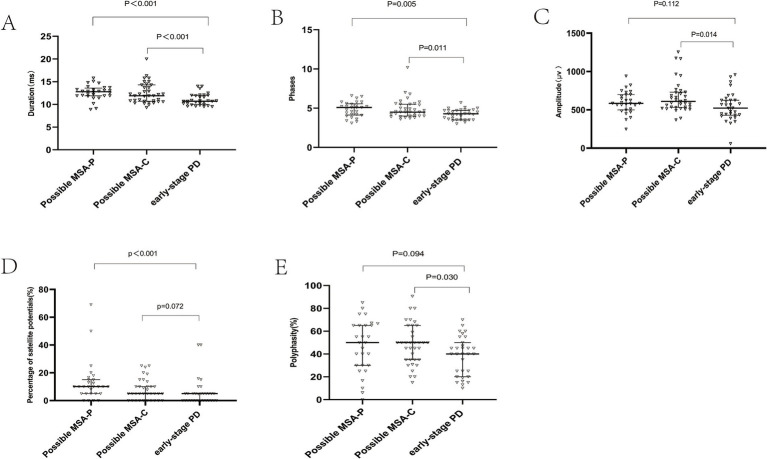
External anal sphincter electromyography findings. Scatter plot of duration **(A)**, phase **(B)**, amplitude **(C)**, satellite potential occurrence rate **(D)**, and polyphase wave ratio **(E)** between MSA-P and PD. This scatter plot depicted the median and interquartile range. Long horizontal lines represent median values.

Previous studies suggested that a cut-off value for mean motor unit action potential (MUAP) duration ranging from 10.9 ms to 14 ms offers the best diagnostic accuracy for MSA. In this study, MUAP duration was significantly longer in possible MSA-P and MUAP duration and phase were significantly longer in possible MSA-P and MSA-C patients compared to early-stage PD patients (Duration Median: possible MSA-P 12.8ms; possible MSA-C 11.9ms; early-stage PD 10.7ms, p<0.001) (Figure 2A); (Phase Median: possible MSA-P 5.10, *p* = 0.005; possible MSA-C 4.5, *p* = 0.011) (Figure 2B). The percentage of satellite potentials was higher in possible MSA-P compared to PD patients (*p* < 0.001) (Figure 2D). Amplitude and polyphasity% were both increased in possible MSA-C patients compared to early-stage PD patients (*p* = 0.014, *p* = 0.011, respectively) (Figures 2C,E).

### 3.3 ROC analysis of PVR urine volume and other autonomic dysfunction indicators

**Figure 3:**
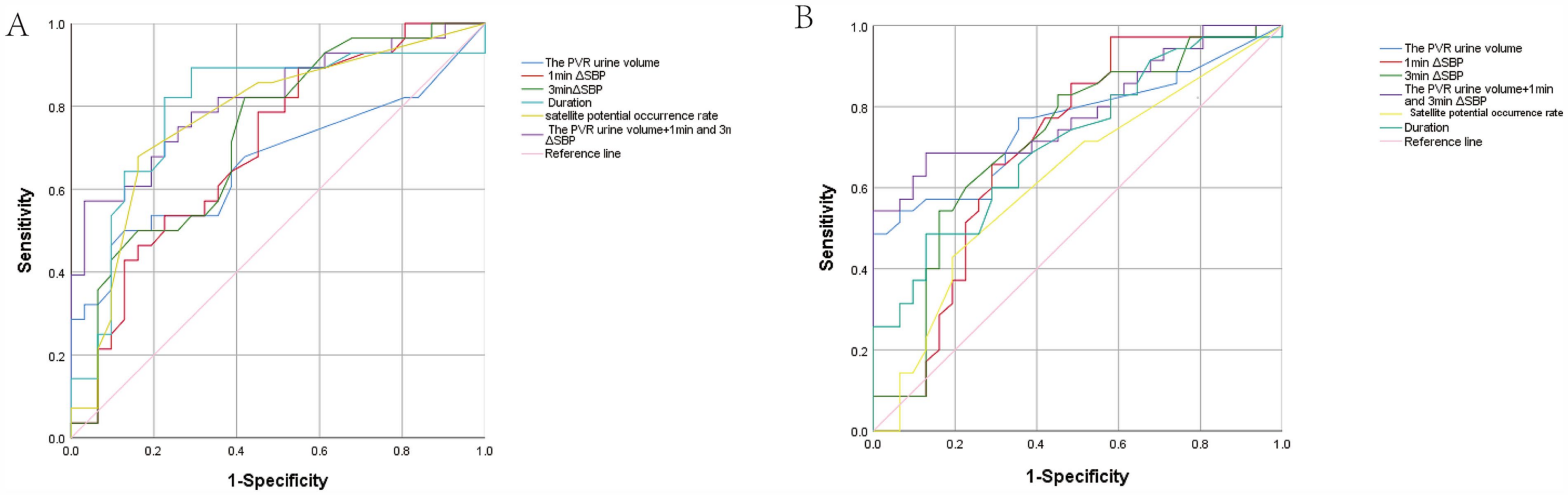
Receiver operating characteristic (ROC) curve analysis. **(A)** ROC analysis of the PVR urine volume and other autonomic dysfunction indicators between possible MSA-P and early-stage PD patients. The blue, red, emerald green, cyan, yellow, purple, and pink represent the PVR urine volume, 1 min ΔSBP, 3 min ΔSBP, Duration, Satellite potential occurrence rate, integrative model and Reference line, respectively. **(B)** ROC analysis of PVR urine volume and other autonomic dysfunction indicators between possible MSA-C and early-stage PD patients. The curves represent the same measures as in **(A)**.

ROC analysis showed that PVR urine volume (AUC = 0.668, a cut-off 24.5 mL), 1-min ΔSBP and 3-min ΔSBP (AUC = 0.702, AUC = 0.730), duration (AUC = 0.797, a cut-off 11.15 ms), and the percentage of satellite potentials (AUC = 0.775, a cut-off 6.5%) had diagnostic value between possible MSA-P and early-stage PD. PVR urine volume, 1-min ΔSBP, 3-min ΔSBP, MUAP duration, and satellite potential occurrence rate discriminated possible MSA-P from early-stage PD with sensitivities of 90.32, 61.29, 51.61, 64.52, and 80.65%, and specificities of 32.63, 60.46, 81.24, 72.80, and 43.94%, respectively. Combining these three indicators improved diagnostic value for possible MSA-P, with an AUC of 0.817, sensitivity of 57.1% and specificity of 96.8%. Notably, combining PVR urine volume with ΔSBP (1 min and 3 min) demonstrated superior ability to differentiate possible MSA-P from early-stage PD compared to EAS-EMG (Figure 3A).

PVR urine volume (AUC = 0.759, a cut-off is 47.50 mL) discriminated possible MSA-C from early-stage PD with a sensitivity of 100% and a specificity of 48.57%. 1-min ΔSBP and 3-min ΔSBP discriminated possible MSA-C from early-stage PD with a sensitivity of 41.94 and 83.87%, and a specificity of 97.14 and 54.29% (1-min ΔSBP: AUC = 0.701, 3-min ΔSBP: AUC = 0.718), respectively. For differentiating possible MSA-C from early-stage PD, the duration (AUC = 0.712, a cut-off is 12.15 ms) yielded a sensitivity of 87.1% and a specificity of 32.99%, while the satellite potential occurrence rate (AUC = 0.624, cut-off 6.35%) with a sensitivity of 80.65% and a specificity of 27.98%. Combining PVR urine volume and ΔSBP (at 1 and 3 min) yielded a AUC of 0.794, sensitivity of 68.6% and specificity of 87.1% (Figure 3B).

### 3.4 Different distribution of α-Syn forms in erythrocytes

**Figure 4:**
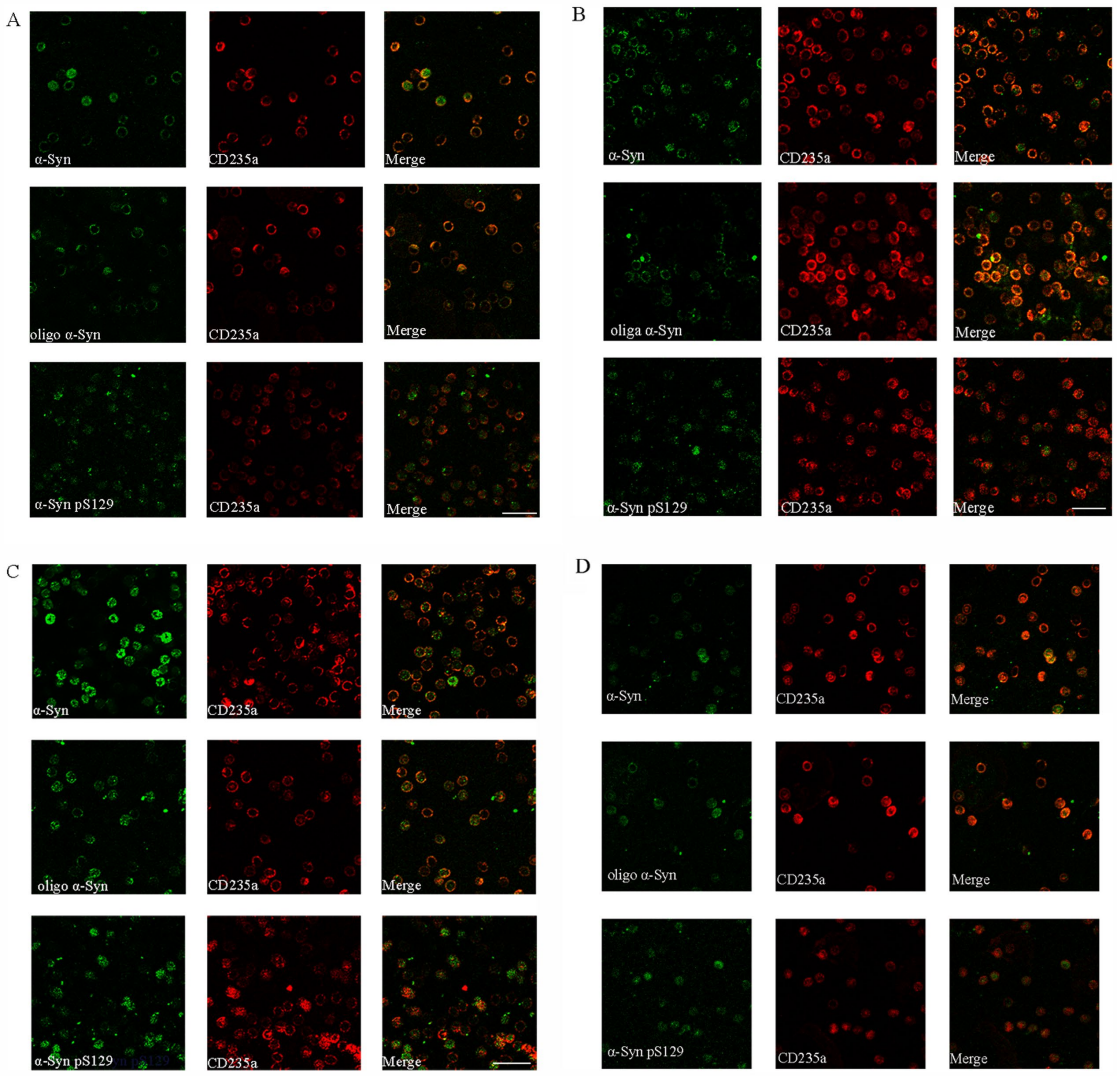
Immunofluorescence staining of α-Synuclein distribution forms in erythrocytes of MSA-P, MSA-C, PD and healthy control (HC) patients. **(A–D)** Representative immunofluorescence images captured by a confocal microscope (40×) Distribution of α-Synuclein (α-Syn), oligomeric α-Synuclein, phosphorylated at Ser129 (pS129) α-Synuclein distribution pattern in erythrocytes of MSA-P **(A)**, MSA-C **(B)**, PD **(C)**, and HC **(D)** groups. The green represents different α-Syn forms. The red represents erythrocyte membrane. Scale bars represent 20 μm in images.

In our study, three different α-Syn forms were stained by immunofluorescence, including α-Syn, Oligo-α-Syn and α-Syn pS129. Immunofluorescence analysis of these α-Syn forms in RBCs revealed that α-Syn was predominantly distributed on the cell membrane fractions, with some presence in the cytoplasm in MSA-P (Figure 4A) and MSA-C patients (Figure 4B). Oligo-α-Syn was mainly observed on the cell membrane in MSA-P and MSA-C patients. In contrast, both α-Syn and oligo-α-Syn were found in the cytoplasm and on the cell membrane in PD patients (Figure 4C) and health controls (Figure 4D). α-Syn pS129 was detected in RBC cytoplasm and membrane fractions in both MSA (MSA-P and MSA-C, Figures 4A,B) and PD patients but was more likely to localize in the cytoplasm in PD patients and health controls (Figures 4C,D). These results suggested a distinct distribution pattern of α-Syn forms in erythrocytes of MSA patients compared to PD patients and healthy controls.

## 4 Discussion

Early and severe autonomic dysfunction is a critical clinical feature of MSA, with bladder dysfunction being a common and early manifestation. Bladder dysfunction in MSA primarily presents as urgency, frequency, weak urinary stream, incontinence, and retention. In advanced stages, catheterization may be necessary, significantly impacting quality of life (Richard et al., 2019). The novel Movement Disorder Society (MDS) criteria for MSA diagnosis include PVR urine volume ≥ 100 mL as an important indicator for clinically established MSA. In clinical practice, PVR urine volume is an effective and objective measure for assessing bladder emptying dysfunction. Investigating bladder dysfunction may provide insights into the pathogenesis of MSA.

In healthy individuals, PVR urine volume should be less than 5 milliliters. The presence of residual urine suggests inadequate bladder function compensation. Previous studies indicate that lower urinary tract symptoms in PD patients typically occur 4–6 years after motor symptom onset, with an incidence of 27–89%. In contrast, 60% of MSA patients exhibit lower urinary tract symptoms before or at the time of motor dysfunction, and nearly 18.2% present with it as the initial symptom (Vichayanrat et al., 2021). Yang-Hyun Lee et al. conducted detailed evaluations of urinary dysfunction between MSA and PD patients, demonstrating a higher PVR urine volume in MSA patients (Lee et al., 2018). Consistent with previous research, our study revealed higher PVR urine volumes in both MSA-P and MSA-C patients compared to PD patients (140.33 [8.25–159.25] ml and 99.52 [11.5–149.5] ml vs. 5 [3.50–20.00] ml, *p* < 0.001). Further analysis confirmed that PVR urine volume remained significantly higher in possible MSA-P and possible MSA-C compared to early-stage PD. This highlights the reliability and objectivity of PVR urine volume as an indicator and emphasizes its role in differentiating MSA from other neurodegenerative disorders, especially in early stages.

While some researchers suggest that MSA-P patients experience a higher incidence and severity of lower urinary tract symptoms due to impaired bladder contraction (Xing et al., 2020), others have shown that MSA-C patients also exhibit a high prevalence of autonomic dysfunction, increased residual urine volume, decreased maximum urinary flow rate, and a higher rate of urinary failure (Kim et al., 2015). Our study found no significant difference in residual urine volume between MSA-P and MSA-C patients. As expected, the duration and occurrence rate of RBD were higher in MSA patients compared to PD patients, likely due to differences in the pathological progression of these neurodegenerative diseases.

Autonomic dysfunction in MSA encompasses urinary tract problems, cardiovascular autonomic dysfunction, thermoregulation disorders, and sexual dysfunction (Sakakibara et al., 2023). Clinically, the STS test is commonly used for rapid assessment of autonomic function in MSA. Over 60% of MSA patients exhibit orthostatic hypotension upon active standing (Jiang et al., 2023). While approximately 20–60% of PD patients may also experience orthostatic hypotension, it typically occurs in later stages (Fanciulli et al., 2020). Our findings demonstrated that both ΔSBP and ΔDBP (at 1 and 3 min) were significantly greater in MSA-P and MSA-C patients compared to PD patients. Similar results were observed between possible MSA-P or possible MSA-C and early-stage PD patients, indicating that cardiovascular autonomic dysfunction is more pronounced in MSA than in PD, even in early stages.

EAS-EMG has been proposed as an ancillary investigation for MSA diagnosis (Todisco et al., 2023). However, the criteria for establishing electrophysiological abnormalities and its true diagnostic value remain debated (Qiu et al., 2019; Vodusek, 2001). Research indicates that the incidence of neurogenic changes in the external anal sphincter within the first year of MSA is 52%, increasing to 83% by the fifth year (*p* < 0.05), suggesting a time-dependent neurodegeneration of Onuf’s nucleus (Todisco et al., 2023). Some studies consider a mean MUAP duration >10 ms as a supportive criterion for MSA diagnosis (Qiu et al., 2013). However, other criteria for neurogenic damage exist. Our results showed that duration, phases, polyphasity, and the percentage of satellite potentials in the anal sphincter were significantly increased in MSA-P and MSA-C patients compared to PD patients. Furthermore, MUAP duration was increased in possible MSA-P and possible MSA-C compared to early-stage PD, and the satellite potential occurrence rate was higher in possible MSA-P compared to early-stage PD. No significant difference in amplitude was observed between possible MSA and early-stage PD.

Previous research suggests that a residual urine volume greater than 150 mL is more helpful than other urodynamic parameters in differentiating MSA from PD (Yamamoto et al., 2017). To improve diagnostic accuracy and establish a suitable preliminary screening protocol for clinical application, we combined PVR urine volume with ΔSBP (at 1 and 3 min). Our ROC analysis revealed that this combination effectively distinguishes possible MSA-P and possible MSA-C from early-stage PD, with AUCs of 0.817 and 0.794, respectively, and a sensitivity of 57.1 and 68.6%, and specificity of 96.8 and 87.1% in both cases. In comparison, the AUC for mean MUAP duration was 0.712 in possible MSA-C and 0.797 in possible MSA-P. The combined PVR and ΔSBP assessment offers a rapid, non-invasive screening method with improved diagnostic accuracy compared to EAS-EMG, which requires more complex skills and can be uncomfortable for patients. These findings align with MDS recommendations for MSA diagnosis and may contribute to the differential diagnosis of early-stage MSA and PD.

A previous study demonstrated that RBCs and dopaminergic cells in the substantia nigra share similar SNCA transcription regulatory factors, potentially leading to a parallel increase in α-Syn (Scherzer C.R et al., 2008). Another recent research found that patients with MSA had greater phosphorylated α-Synuclein deposition and more widespread peripheral distribution in comparison with PD patients (Gibbons et al., 2023). There was a significant positive correlation of erythrocytic aggregated α-Syn concentrations with disease duration of essential tremor (Yu et al., 2023). In our previous study, it found that the RBC α-Syn levels was positive with longitudinal rates of clinical progression in early PD (Liu et al., 2022). To date, the relationship of blood-based α-Syn levels and motor symptom progression were still inconclusive (Niu et al., 2020; Lee et al., 2006). Therefore, direct observation of α-Syn distribution in erythrocyte is helpful to explore different synuclein diseases procession.

Our previous research showed increased levels of oligomeric α-Syn/total α-Syn and oligomeric α-Syn/protein in the erythrocyte membrane of MSA patients compared to healthy controls (Liu et al., 2019). Consistent with these observations, the present study found that α-Syn and oligo-α-Syn were mainly distributed on the cell membrane fractions in MSA patients, with some presence in the cytoplasm. In contrast, both were found in the cytoplasm and on the cell membrane in PD and health control patients. α-Syn pS129 was detected in the cytoplasm and membrane fractions in both MSA and PD patients but was more likely to localize in the cytoplasm in PD and health control patients. Above these observations, we found that the distribution of α-Syn forms on the cell membrane fractions was more common in MSA patients than PD or health control patients. The pathogenic mechanisms underlying the diverse distribution of erythrocyte α-Syn forms in synucleinopathies remain unclear. We speculate that the localization of toxic α-Syn species primarily in the erythrocyte membrane of MSA patients may contribute to cell membrane disruption and cellular dysfunction. Future studies should investigate the link between α-Syn forms and autonomic dysfunction.

## 5 Limitations

This study has limitations. Firstly, a larger sample size is needed to verify the reliability and accuracy of our findings. Secondly, longitudinal observation of PVR urine volume changes in both MSA and PD patients is necessary to provide further evidence. Thirdly, while this study focused on established cases of MSA and PD, the inclusion of healthy controls, iRBD, early-stage MSA and PD patients is essential for understanding the early biomarkers of these disorders, which will be addressed in future studies. Finally, in order to ensure consistency for inclusion criteria, we adopted the second consensus criteria of MSA in 2008, rather than the latest MSA criteria formulated by Movement Disorders Society Scientific Issues Committee in 2022, and the differences between these criteria need to be explored in future studies.

## 6 Conclusion

Early MSA diagnosis is crucial to avoid unnecessary urological interventions and prevent potentially severe complications. PVR urine volume sonography combined with the STS test may be a suitable screening method for differentiating MSA from PD, even in early stages. The distribution patterns of distinct α-Synuclein forms in erythrocytes are also valuable for differential diagnosis.

## Data Availability

The original contributions presented in the study are included in the article/supplementary material, further inquiries can be directed to the corresponding authors.
